# Supplementing routine hospital surveillance of malaria to capture excess mortality and epidemiological trends: a five-year observational study

**DOI:** 10.3389/fmala.2024.1340276

**Published:** 2024-04-16

**Authors:** Jean-Bertin Bukasa Kabuya, Caitlin Bond, Manuela Hauser, Jay Sikalima, Bruce Phiri, Dickson Phiri, Japhet Matoba, Jayme Hughes, Proscovia Miiye Banda, James Sichivula Lupiya, Gershom Chongwe, Philip E. Thuma, William J. Moss, Matthew M. Ippolito

**Affiliations:** 1 Clinical Sciences Department, Tropical Diseases Research Centre, Ndola, Zambia,; 2 Department of International Health, Johns Hopkins Bloomberg School of Public Health, Baltimore, MD, United States,; 3 Department of Paediatrics, Cantonal Hospital Graubünden, Chur, Switzerland,; 4 Office of Health Management Information Systems, Saint Paul’s General Hospital, Nchelenge, Zambia,; 5 Macha Research Trust, Choma, Zambia,; 6 Department of Molecular Microbiology and Immunology, Johns Hopkins Bloomberg School of Public Health, Baltimore, MD, United States,; 7 Clinical Laboratory, Saint Paul’s General Hospital, Nchelenge, Zambia,; 8 Department of Epidemiology, Johns Hopkins Bloomberg School of Public Health, Baltimore, MD, United States,; 9 Johns Hopkins Malaria Research Institute, Johns Hopkins Bloomberg School of Public Health, Baltimore, MD, United States,; 10 Department of Medicine, Johns Hopkins University School of Medicine, Baltimore, MD, United States

**Keywords:** malaria, *Plasmodium falciparum*, severe malaria, Zambia, disease surveillance

## Abstract

**Introduction:**

Malaria surveillance in Africa is conducted largely through health facility-based health management information systems (HMIS) which provide aggregated data to malaria control programs. Supplementation of HMIS surveillance with other routinely collected hospital data can provide vital statistics on malaria control in regions of high burden.

**Methods:**

To assess the utility of supplementing HMIS data, we implemented a pilot program of enhanced malaria surveillance in a district hospital in northern Zambia over a five-year period. Data were tabulated from existing nursing records, central pharmacy inventories, laboratory logbooks, and ward registers and cross-referenced with routinely collected HMIS data.

**Results:**

The additional data collections captured excess malaria deaths resulting from pharmacy and blood bank stockouts (10.3 excess deaths/year) and revealed small but significant changes over time in the age distribution of patients that likely reflect underlying shifts in the local epidemiology due to malaria control programming or other factors (median age from 1.9 to 2.4 months old, P=0.001).

**Discussion:**

Readily available data can supplement existing HMIS surveillance in high malaria burden areas to provide actionable information about the local epidemiology and impacts of control efforts. Excess malaria deaths due to health systems factors can be feasibly captured and tracked and fed back to national malaria control programs and the World Health Organization to present a fuller picture of malaria burden.

## Introduction

Public health surveillance for malaria commands an integral place in control programming. Today in sub-Saharan Africa, malaria surveillance networks are coordinated largely through national control centers via health management information systems (HMIS) that collate data from clinics, hospitals, and their auxiliaries (World Health Organization, 1988; World Health Organization, 2000). In addition to providing essential information for operational planning, these data are key inputs into regional and global estimations of malaria trends (World Health Organization, 2023). The challenges of gathering reliable statistics in remote, rural parts of Africa is well-documented (Arudo et al., 2003; Alegana et al., 2020; Kigozi et al., 2020), and the firming up of existing surveillance networks has progressed with the standardization of data collection tools and the move from paper-based to electronic reporting and visualization (Wangdi et al., 2020).

In a recent strategic report, the World Health Organization advocates for reinterpreting malaria surveillance as a core intervention, emphasizing that accurate, intelligible, and actionable data are prerequisite to a functioning control program (World Health Organization, 2019). Passive surveillance based out of health facilities captures the local epidemiology and assists with budgeting, procurement, and allocation of resources across geographic areas that can have vastly different malaria endemicities. Passive health facility-based surveillance also is a tool to measure the impacts of control efforts ranging from indoor residual spraying campaigns to insecticide treated net distributions to case management and upcoming malaria vaccine rollouts (Comfort et al., 2014; Ippolito et al., 2022a).

In high-burden areas, malaria surveillance has historically received less attention than in pre-elimination settings where focus is placed on identifying and stymieing outbreaks in near real-time (World Health Organization, 2007; Perera et al., 2020). Rethinking surveillance in high-transmission areas therefore presents an opportunity for heightening the effectiveness of control (World Health Organization, 2019). Prior commentators have suggested incorporating data adjacent to that collected in the formal HMIS structure (Mutemwa, 2006). We observed that facility-based malaria surveillance activities intersect with a number of hospital workflows which, when integrated together, could lend potentially useful information to render the local epidemiology of malaria in richer relief without significantly more effort or capital. By expanding data collection, we sought to identify the minimum essential information that could meaningfully inform conventional malariometrics.

Additionally, we were interested in understanding the impact and relevance of hospital stockouts to malaria surveillance endpoints and how short-term trends in the age distribution of hospitalized patients with malaria might provide an early sign of changing epidemiology in local communities. When chloroquine resistance first appeared on the African continent, one of the earliest signs was an uptick in pediatric blood transfusions, establishing blood banks as de facto early warning systems in some places (Trape et al., 1998). With these objectives, we implemented a suite of supplemental surveillance procedures to HMIS at a district-level hospital in a high transmission region of northern Zambia and here report our findings from the first five years.

## Methods

### Study site

The pilot program was implemented April 2017 in a district-level hospital in northern Zambia’s Luapula Province within the study area of the Southern and Central Africa International Center of Excellence (ICEMR). Data are presented to June 2022. The district is home to a population of over 300,000 people, mostly subsistence farmers and fishers (Sikalima et al., 2021). The predominant malaria parasite is *Plasmodium falciparum*, which circulates year-round at high intensity through *Anopheles funestus* and *An. gambiae* mosquito vectors (Ippolito et al., 2022a). Intensive vector control has been attempted with over a decade of annual indoor residual spraying and multiple insecticide-treated net distributions with little measurable impact (Mukonka et al., 2014; Hast et al., 2019). The hospital is a 175-bed referral center for fifteen rural health centers located 1.5 to 40 km away. Malaria accounts for the plurality of pediatric admissions and deaths (Ippolito et al., 2018). Diagnosis is almost exclusively made by antigen-based rapid diagnostic test; microscopy is scarcely done (Ippolito et al., 2022b). Artesunate has been first-line treatment for patients meeting criteria for severe malaria since 2012, and antibiotics are often given empirically. When artesunate is not available, quinine is administered or, if quinine is contraindicated or also not available, then oral artemisinin-based combination therapy is given.

### Data collection

Routine HMIS data consisted of the number of monthly inpatient and outpatient department visits for malaria and the number of inpatient deaths grouped by age (<1 year, 1 to <5 years, ≥5 years). Analyses were focused on the youngest two age groups. An initial site visit identified four additional sources of routinely collected data for which data capture forms were developed. (1) In the children’s ward a nursing medication administration record documented individual patients receiving artesunate. From these forms, individual-level data were recorded consisting of the date of administration and patient age. (2) From the blood transfusion logbook in the clinical laboratory, the number of pediatric blood transfusions was counted and for each record the age of the patient, date of transfusion, indication, and hemoglobin concentration were entered. (3) A handover logbook recorded the numbers of units of blood in the blood bank at the start of each laboratory shift. Blood inventory was recorded as the number of units of type A, B, AB, and O blood in stock each morning. (4) Artesunate inventory was similarly recorded from the central pharmacy as the number of units in stock and the number of units dispensed to the wards. Existing HMIS staff were trained to collect the supplemental data and transmit it monthly to the investigators who processed it for entry into a central database using Research Electronic Data Capture (Harris et al., 2009). Pharmacy records were unavailable for 4 months in 2018 and 2 months in 2019, and blood inventory data were missing for 7 months in 2017–2018 and 4 months in 2021 due to missing or destroyed records. Hospital catchment area population estimates were obtained from Geo-Referenced Infrastructure and Demographic Data for Development (Borkovska et al., 2022).

To estimate excess deaths due to blood stockouts, individual-level data from pediatric patients with severe malarial anemia, defined as a chart diagnosis of malaria and hemoglobin concentration ≤5 g/dL, were collected from ward registers over a 21-month interval during the study period (n=722). To externally validate the HMIS data and to describe the background epidemiology of malaria in the community, data were collected from a short message service-based health center surveillance system (Shiff et al., 2013) and existing community cohort under the ICEMR (Ippolito et al., 2022a), and from the Zambian District Health Information System (DHIS). Ethical approvals were obtained from the Johns Hopkins Bloomberg School of Public Health Institutional Review Board and the Tropical Diseases Research Centre Ethics Review Committee.

### Data interpolation

From January to April 2018, clinic and hospital cases diverged due to a reporting bias caused by the resettlement of Congolese refugees to the study area (Hauser et al., 2023). Clinic case data excluded the refugee clinic while hospital surveillance captured refugee and non-refugee cases alike. To interpolate monthly refugee clinic cases, we divided the number of refugee hospital cases, estimated based on review of ward registers to be 12% of total pediatric malaria admissions for that period, by the expected proportion of health clinic cases that proceed to severe malaria (2%).

### Statistical analysis

Means and standard deviations were calculated and compared in unadjusted univariable analyses using Student’s t test and oneway analysis of variance. Dichotomous outcomes for patient-level data were analyzed using Pearson’s chi-squared test. Comparisons of malaria metrics were done by comparing each metric to HMIS admission data and plotting them against the line of equality. Stockouts of artesunate and whole blood were considered both dichotomously (stockout lasting ≥1 week) and continuously (number of days of stockout) and tested for statistical significance in linear regression models of case fatality on stockout. Individual-level data were analyzed in logistic regression models of mortality on stockouts adjusted for age, sex, and hemoglobin concentration. Sensitivity analyses excluding the January–April 2018 period were done. Analyses were done in Stata 17 MP (StataCorp, College Station, Texas, USA) and R 4.2.0 (R Foundation for Statistical Computing, Vienna, Austria).

## Results

**FIGURE 1:**
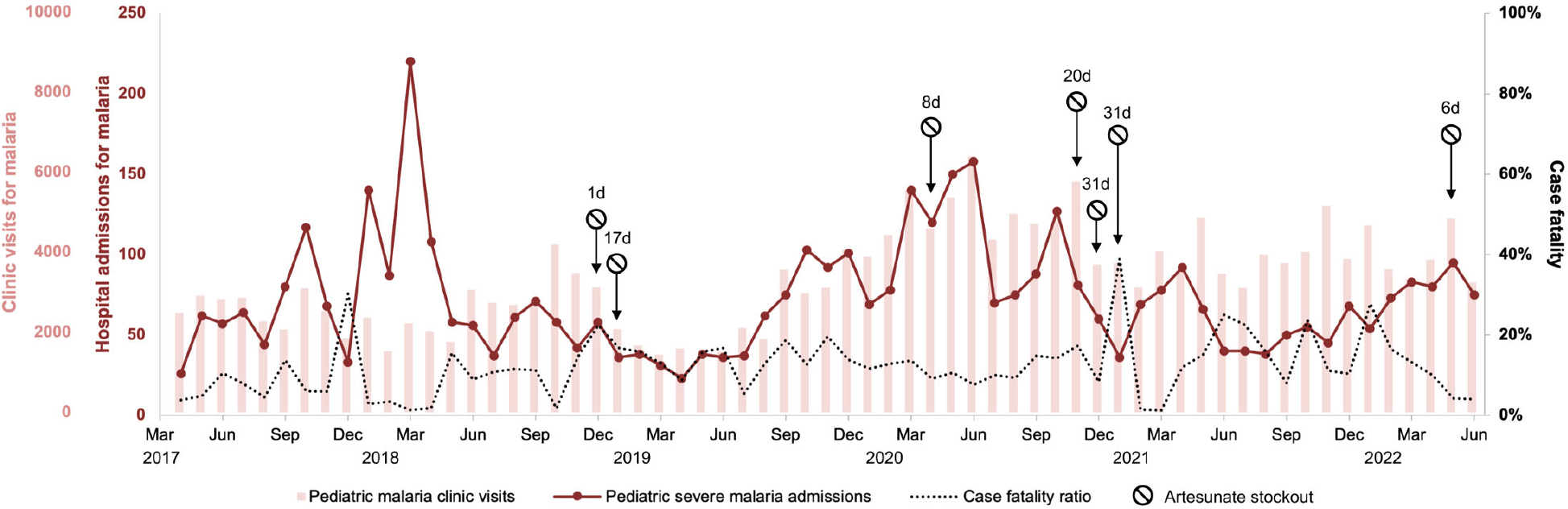
Trends over time of malaria clinic visits, hospital admissions for malaria, and in-hospital case fatality in children under 5 years old. The discrepancy between clinic visits and hospital admissions in early 2018 is attributable to an influx of Congolese refugees into the study area which were captured by the hospital surveillance data but not clinic surveillance data. Duration of artesunate stockouts shown in days (d).

**FIGURE 2:**
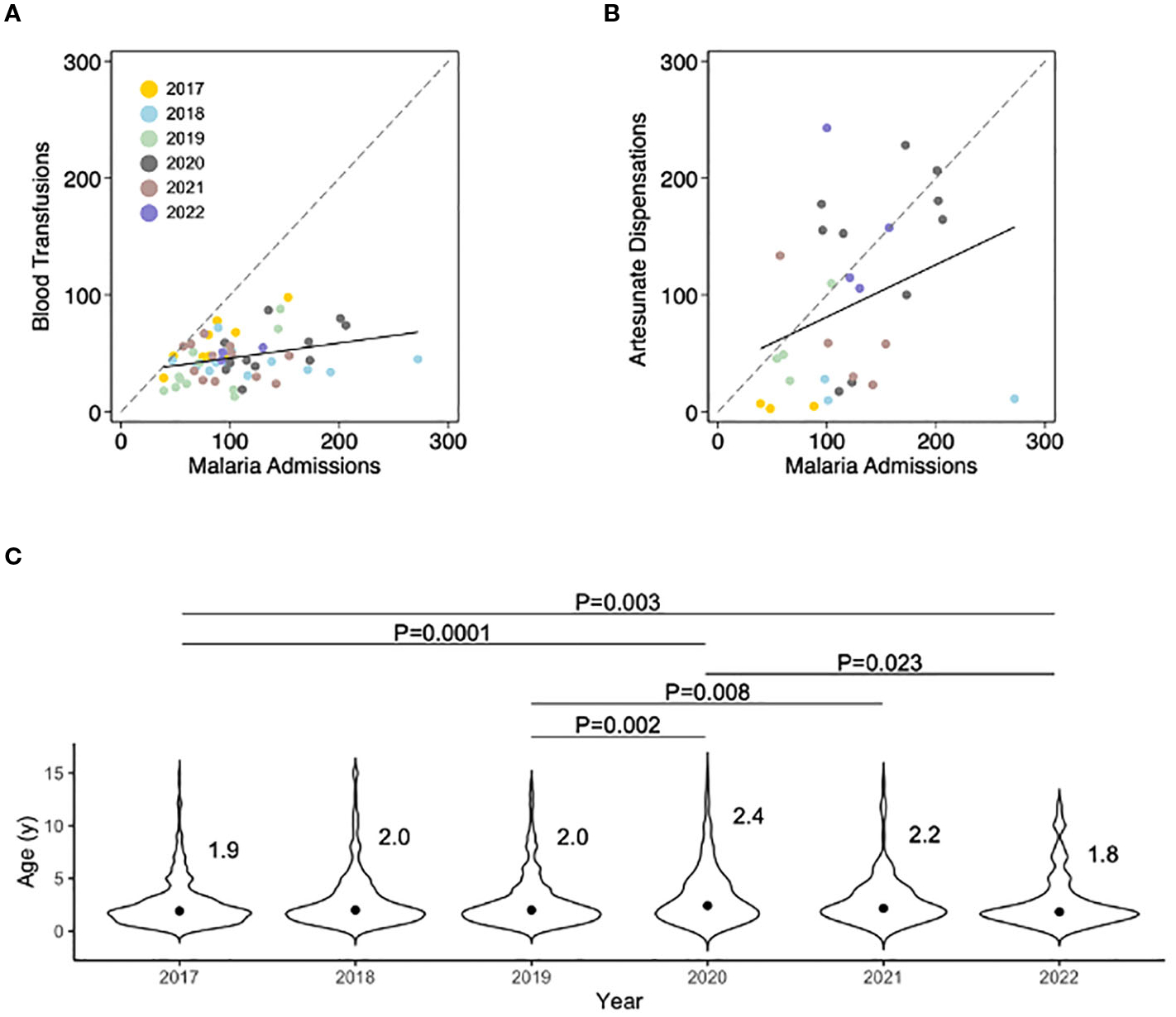
Comparisons between the number of monthly malaria admissions recorded in the Hospital Management Information System with the number of pediatric blood transfusions and artesunate dispensations, and year-on-year trends in the median age of pediatric patients with severe malaria. **(A)** Monthly number of pediatric blood transfusions plotted against HMIS case data (correlation coefficient 0.34, P=0.01). **(B)** Monthly number of units of artesunate issued by the hospital central pharmacy plotted against HMIS case data (correlation coefficient 0.37, P=0.05). **(C)** Subtle but real shifts in the age distribution of hospitalized children with malaria can provide early information about the changing epidemiology of malaria and population immunity. Here, individual patient ages were gathered from existing nursing documentation. The distribution around the median is shown and was compared using the Kruskal-Wallis test. Importantly, between 2018–2019 an intensive program of integrated community case management with a reactive test-and-treat component was implemented and then discontinued. Annual indoor residual spraying was conducted before the onset of the rainy season in all years and net coverage hovers around 40% according to community surveys.

During the study period, there were 192,779 cases of malaria in children under 5 years old within the hospital catchment area leading to 4,244 (2.2%) hospital admissions with an inpatient case fatality ratio of 11% (Figure 1). Annual malaria incidence was estimated to be 896 clinical cases, 19 hospitalizations, and 2 deaths per 1,000 population under 5 years old. The number of pediatric blood transfusions and artesunate dispensations from the hospital pharmacy were both significantly correlated with HMIS malaria admission data (Figures 2A, B). We found that medication administration records were used by nursing only during periods of high patient volume, and therefore did not accurately track long-term HMIS admissions data.

The median age of pediatric patients treated with artesunate was similar to that of pediatric patients who underwent blood transfusion (median 24 months, interquartile range 14–43), and across the five years of surveillance, statistically significant fluctuances in the median age were seen with steadily increasing age over the period 2017–2020 which then fell in 2021–2022 (Figure 2C).

**FIGURE 3:**
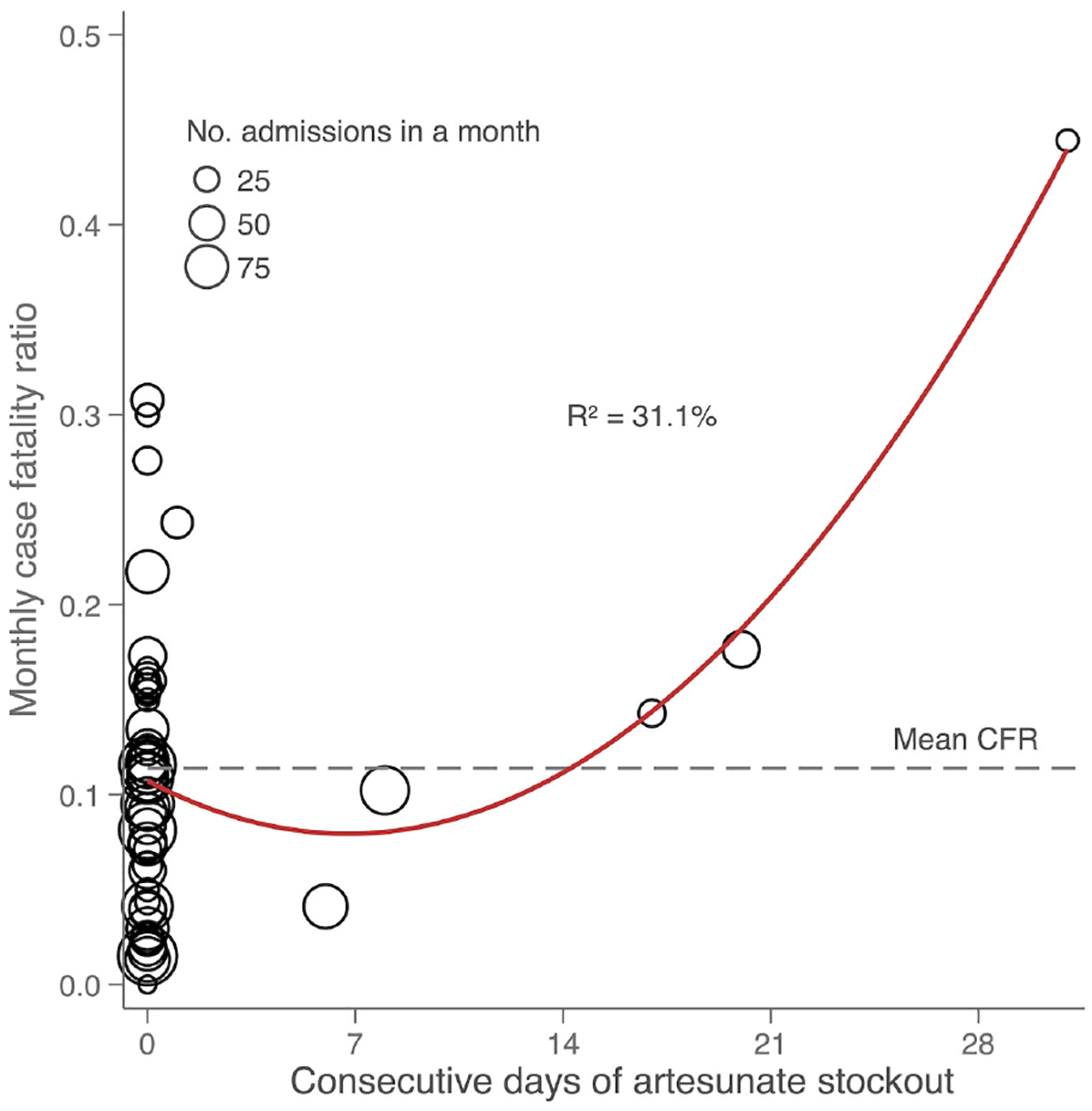
Scatterplot showing the relationship between monthly case fatality and consecutive days of artesunate stockout in hospitalized children 1 to <5 years old with a diagnosis of malaria. The dashed line represents the average monthly case fatality ratio (CFR) and the R^2^ value is that of the quadratic line of best fit.

There were six months with at least one artesunate stockout lasting from 1 day up to 31 days. Malaria case fatality in children under 5 years old during months with ≥1 week of artesunate stockout was 18% compared to 11% in months with no stockout or stockout lasting <1 week (P=0.07). The difference was mainly in children 1 to 5 years old (19 vs 11%, P=0.03); case fatality in patients >5 years old did not differ (Table 1). For each consecutive day of artesunate stockout in a month, there was a 0.67 percentage point increase in monthly malaria case fatality in children 1 to 5 years old (95% CI 0.29–1.06, P=0.001, adjusted for year, month, and concomitant blood stockout) (Table 1 and Figure 3). This translated to 21 excess malaria deaths in children 1 to 5 years old during the 45 months for which all data were available (5.6 excess deaths/year). Sensitivity analysis excluding the first four months of 2018 showed the association between artesunate stockouts and mortality was preserved.

Blood stockouts, either partial or complete for durations of 1 week or longer, did not correlate significantly with the monthly case fatality ratio for malaria in either unadjusted or adjusted models. However, in children with severe malarial anemia, blood stockouts were positively correlated with mortality. Severe malarial anemia was present in 50% of children under 5 years old with a diagnosis of severe malaria. Case fatality in this group was 19% overall. For those admitted on days when matching blood was not available, case fatality was significantly higher than for those admitted on days when blood was available (25 vs. 15%, Pearson’s chi-squared P=0.033). Stockouts of the matching blood group type or types (e.g., type A or O for patients with blood group type A) nearly doubled the odds of death (odds ratio 1.9 adjusted for age, sex, and hemoglobin, 95% CI 1.03–3.44, P=0.041). Blood stockouts impacted 91 out of 310 (29%) children under 5 years old admitted with a diagnosis severe malarial anemia, resulting in 10 excess deaths over the 21-month period for which data were available (5.7 excess deaths/year).

We hypothesized that increased hospital admissions for malaria would lead to stockouts, but neither artesunate nor blood stockouts were associated with patient volume. An unexpected correlation, however, was seen between patient volume and monthly malaria case fatality. Case fatality was inversely correlated with patient admissions: controlling for artesunate stockouts, for every 100 hospital admissions for malaria the monthly case fatality was 7.0 percentage points lower (95% CI 2.0–12.0, P=0.007).

## Discussion

A five-year pilot program of supplemented HMIS surveillance for malaria yielded information about the local epidemiology and allowed estimation of excess mortality that routine HMIS data does not capture. The background epidemiology of malaria was typical for a high-transmission settings (World Health Organization, 2014): for children under 5 years old, a minority of cases in the community progress to severe disease requiring hospitalization (~2%) which carried a risk of fatality of 11%.

Artesunate stockouts resulted in excess deaths in children under 5 years old that were measurable from the monthly aggregated data. Every consecutive day of a stockout led to a 5% increase in case fatality. The higher mortality likely stems from suboptimal treatments with quinine or oral therapy in lieu of intravenous artemisinin, which has been shown in clinical trials to be superior to alternatives (Dondorp et al., 2010). Artesunate stockouts have previously been implicated as a recurring challenge to control programs (Ojo et al., 2020); here, we show the ease with which the data can be collected and modeled to estimate excess deaths.

Pediatric blood transfusions systematically tracked with malaria hospital admissions, as has been previously shown under conditions of moderate to high transmission (Comfort et al., 2014). The impact of blood stockouts on malaria mortality was not detectable in the aggregated HMIS data, which does not distinguish cases of severe malarial anemia from other clinical presentations of malaria. In the patient-level data, however, blood stockouts of matching blood types were shown to be associated with nearly double the risk of mortality in children with severe malarial anemia, a similar finding to our previously published results of a retrospective study of blood transfusion for severe malarial anemia at the same site (Ippolito et al., 2022b). In practical terms, the effort required to account for blood group type of the units of blood in the blood bank and the individual patients presenting with severe malarial anemia is outside the range of minimum essential data for current surveillance, which still relies largely on paper records.

Stockouts of essential medicine and blood were not associated with patient volume, pointing to more fundamental issues in supply chain management. Mobile technology, cold chains, information technology, and upskilling of individuals throughout the supply chain are some of the footholds for malaria control programs to begin horizontal integration of health systems logistics (Lewis et al., 2012; Ansah et al., 2022). Similarly, a linked electronic medical record system would streamline if not obviate the need for assembling an enhanced malaria surveillance system from existing hospital workflows. Urban areas in Zambia and elsewhere throughout Africa have made the switch, but progress is slower in the remote, rural hospitals where malaria is most entrenched.

In addition to quantifying the extent of excess malaria deaths due to health systems issues, adding routinely collected data from nursing documents to existing HMIS surveillance helped elaborate the underlying epidemiology of malaria over time. The ancillary nursing and transfusion data allowed analysis of age trends that HMIS protocols do not accommodate (HMIS data aggregates data according to age groups). The changes over time in the median age of patients hospitalized with malaria likely signal shifts in the background transmission intensity of malaria (Snow et al., 1997) due to control interventions, impacts of the Covid pandemic, or other factors as has been observed in related studies of malaria hospital epidemiology with more demanding data collection (Idro et al., 2006; Guinovart et al., 2022).

In this way, the enhanced surveillance sought to capture the evolving epidemiology of malaria in an area of intense transmission undergoing correspondingly intense control efforts with widescale insecticide-treated net distributions, annual indoor residual spraying campaigns, case management with rapid diagnostic tests and artemisinin-based combination therapy, and integrated community case management. We speculate that these small but real fluctuations in the age distribution of children hospitalized for malaria reflect short-term impacts of control efforts, as community reductions in malaria decelerates the rate of immunity acquisition.

Similarly, the unexpected association between patient volume and case fatality—malaria case fatality was highest during ebbs of hospital admission, and lowest during peak malaria months—is puzzling, and might point to a similar phenomenon. Whether this pattern is observed in other highly malarious areas would be important to determine because of its implications for malaria control. Possibly, the pattern is due to waxing and waning premunition throughout the year as exposure to parasites varies according to weather and the predominant mosquito vector (Sergent and Parrot, 1935). If this were so, it raises the specter of rebound malaria (Graboyes and Meta, 2022): if short-term changes in protective immunity occur to an extent that causes observable changes to malaria case fatality over a term of just months, it reinforces the need for malaria control programming to include contingencies for upticks in severe cases resulting from falling immunity and to ensure a plan for sustaining any gains that are realized. The possibility remains that it is an artifact of data collection or misclassification bias, which stalks all epidemiological studies of severe malaria in high-transmission regions (Ippolito and Robinson, 2022). Going forward, flow cytometry for automated confirmation of malaria will be implemented in the hospital (Kagaya et al., 2022) as a use case for teasing out falsely or incidentally positive rapid antigen tests or microscopy results, which are suspected of occurring in up to one-third of hospitalized severe malaria cases (Bejon et al., 2007). Refining the case definition of severe malaria is critical to increasing the confidence with which these and other associations are ascribed, and progress thereby steered in the right direction.

There is value in collecting only the minimum essential data. We tested the range of what should be considered minimally essential by supplementing a conventional HMIS malaria surveillance system and assessing whether useful data could be gained from the added effort. Early detection of shifts in malaria epidemiology by tracking the age distribution of malaria hospitalizations could have use for predicting rebound in the setting of a vaccine rollout, for example, or gauging the impact of control interventions in high-transmission settings. The simple step of cross tabulating HMIS data with pharmacy inventories allows excess, avoidable malaria deaths to be calculated, providing important feedback to national programs. This is a metric that should be estimated and tracked in the World Health Organization’s World Malaria Report, quantifying the cost in lost lives of supply chain management shortfalls and, conversely, the opportunity for reducing malaria burden by addressing those shortfalls.

There are three current trends in malaria control that provide important context for this study: surveillance as a core intervention, subnational tailoring of malaria control programming, and the horizontal integration of malaria control activities into existing health systems (World Health Organization, 2022). This pilot program demonstrates that with little additional effort or cost, existing data can feasibly be gathered at sentinel hospitals and incorporated into malaria surveillance to provide a more complete, dynamic picture of the local epidemiology. There are also interventions on the horizon—vaccines, monoclonal antibodies, long-acting injectables—that could dramatically alter malaria epidemiology in places that have been historically recalcitrant to the conventional methods. Now is the time to conceive of and test suitably informative surveillance approaches so that their impacts can be anticipated and monitored.

## Figures and Tables

**TABLE 1:** Inpatient malaria case fatality by age group and artesunate stockout.

Age group	Monthly average case fatality (%)		P value	Mean point increase in monthly CFR per consecutive stockout day		P value
	No artesunate stockout	Artesunate stockout*		β	95% CI	
<1 year old	13.0 ± 10.2	17.3 ± 12.1	0.401	0.37	−0.12–0.87	0.139
1 to <5 years old	11.0 ± 7.4	19.3 ± 14.4	0.032	0.67	0.29–1.1	0.001
>5 years old	11.8 ± 7.5	13.3 ± 5.2	0.652	0.09	−0.27–0.45	0.616

* Artesunate stockout lasting ≥1 week. Means ± standard deviations are shown. CI, confidence interval. P values calculated by Student’s t test or multilinear regression. Multilinear regression adjusted for year, month, and concomitant blood stockout.

## Data Availability

The raw data supporting the conclusions of this article will be made available by the authors, without undue reservation.

## Highlights

- Redefining the minimum essential data for malaria surveillance can help address current gaps in malaria reporting.
- Hospital-based sentinel surveillance of malaria that incorporated readily available clinical, laboratory and pharmacy data was piloted in an area of intense transmission in Zambia.
- This approach enabled estimation of excess malaria mortality and subtle but significant shifts in malaria epidemiology.
